# Supplementary figures and images for: m6A Methylation Regulators Are Predictive Biomarkers for Tumour Metastasis in Prostate Cancer
Source: Cancers (Basel). 2022 Aug 21;14(16):4035. doi: 10.3390/cancers14164035 (PMC9406868; doi:10.3390/cancers14164035)

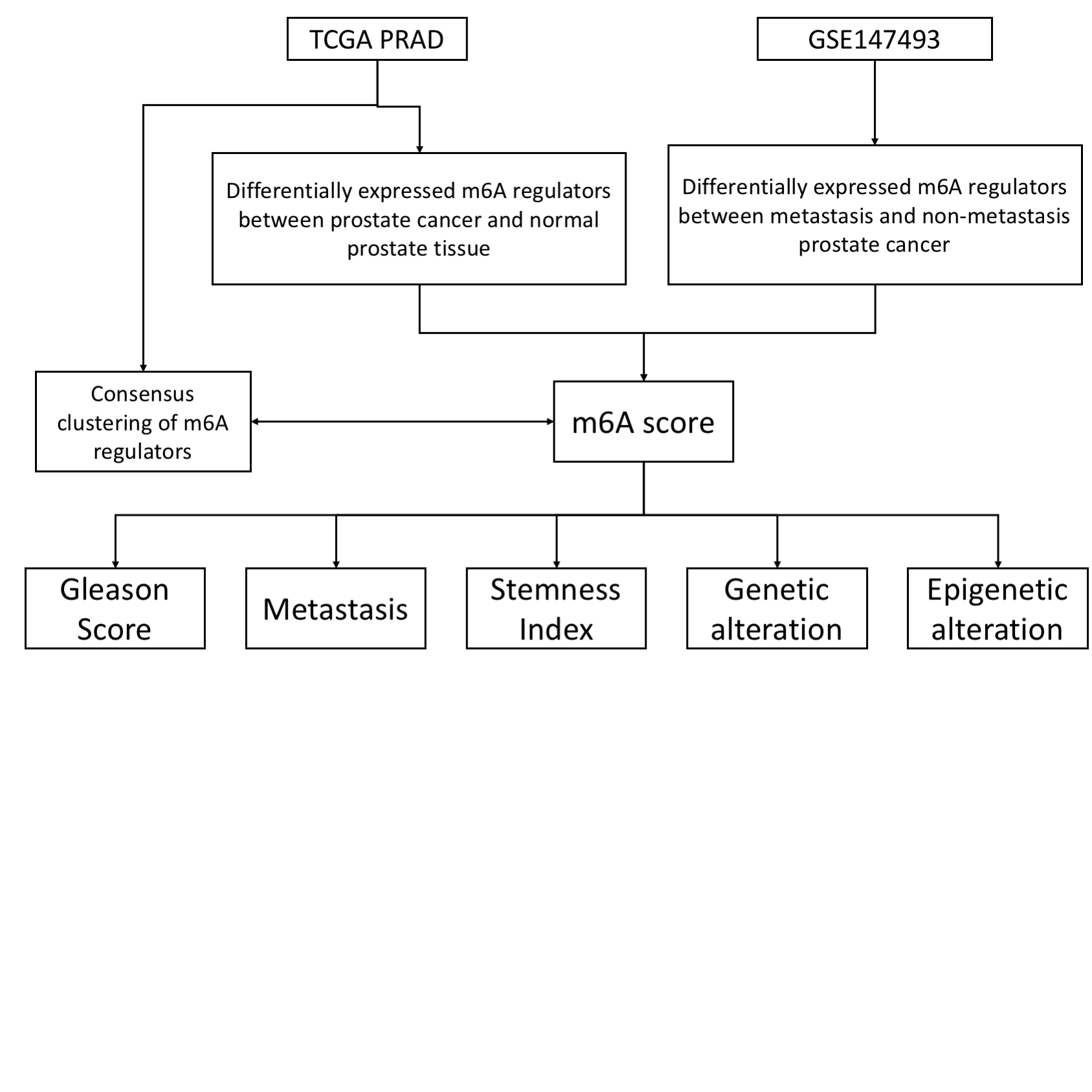

Supplement: Supplementary file 1 [file cancers-14-04035-s001.zip › Supplementary Figure S1.tiff]

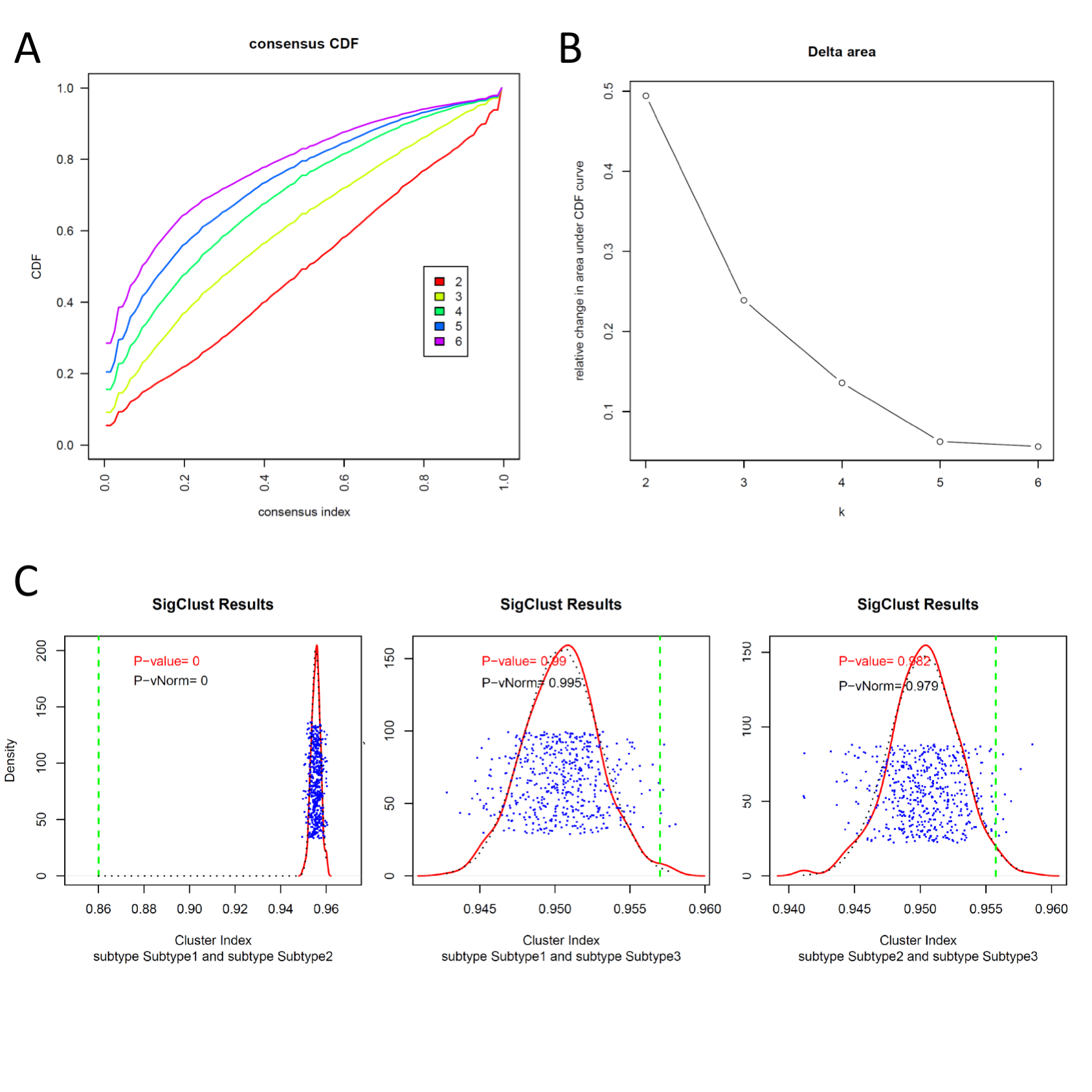

Supplement: Supplementary file 1 [file cancers-14-04035-s001.zip › Supplementary Figure S2.tiff]

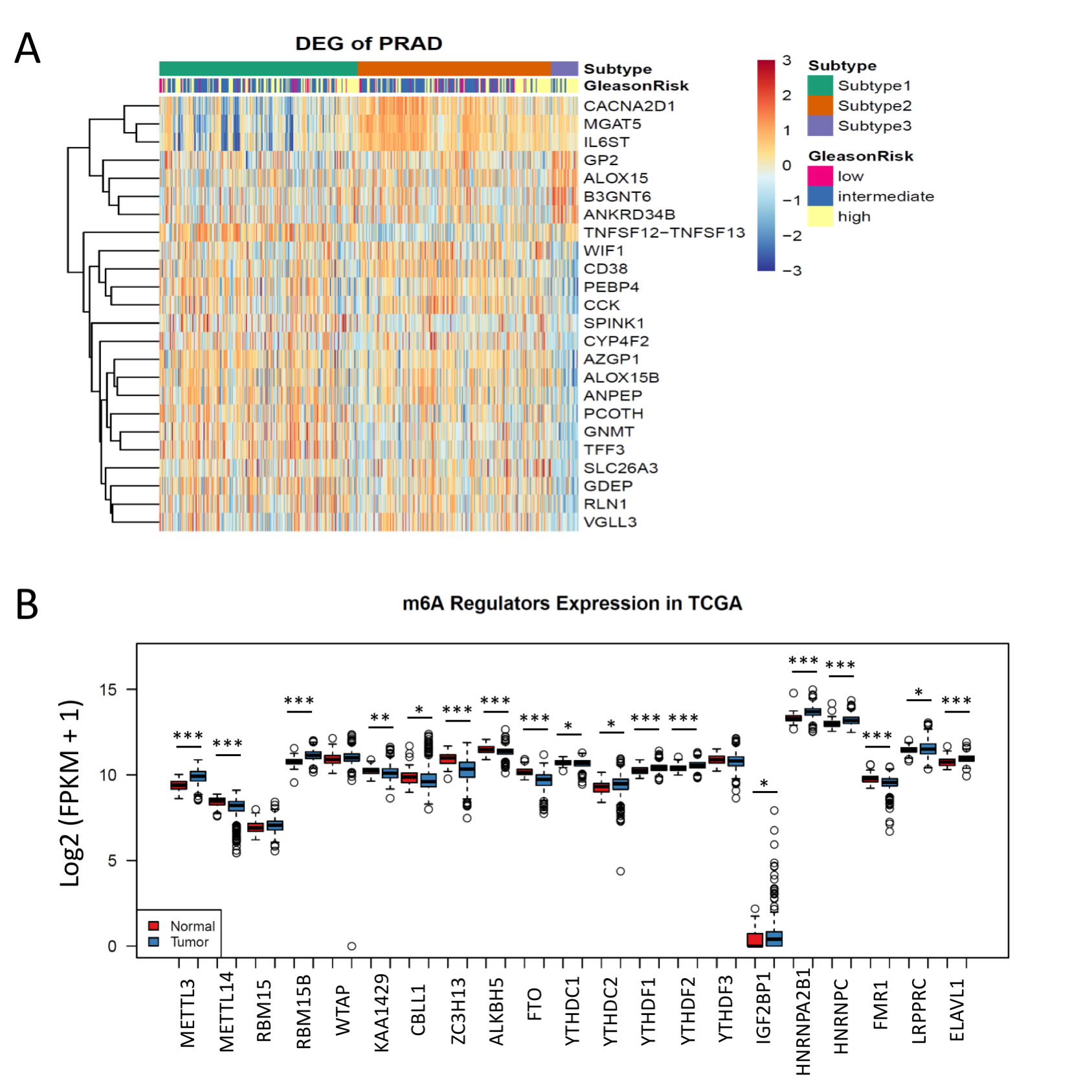

Supplement: Supplementary file 1 [file cancers-14-04035-s001.zip › Supplementary Figure S3.tiff]

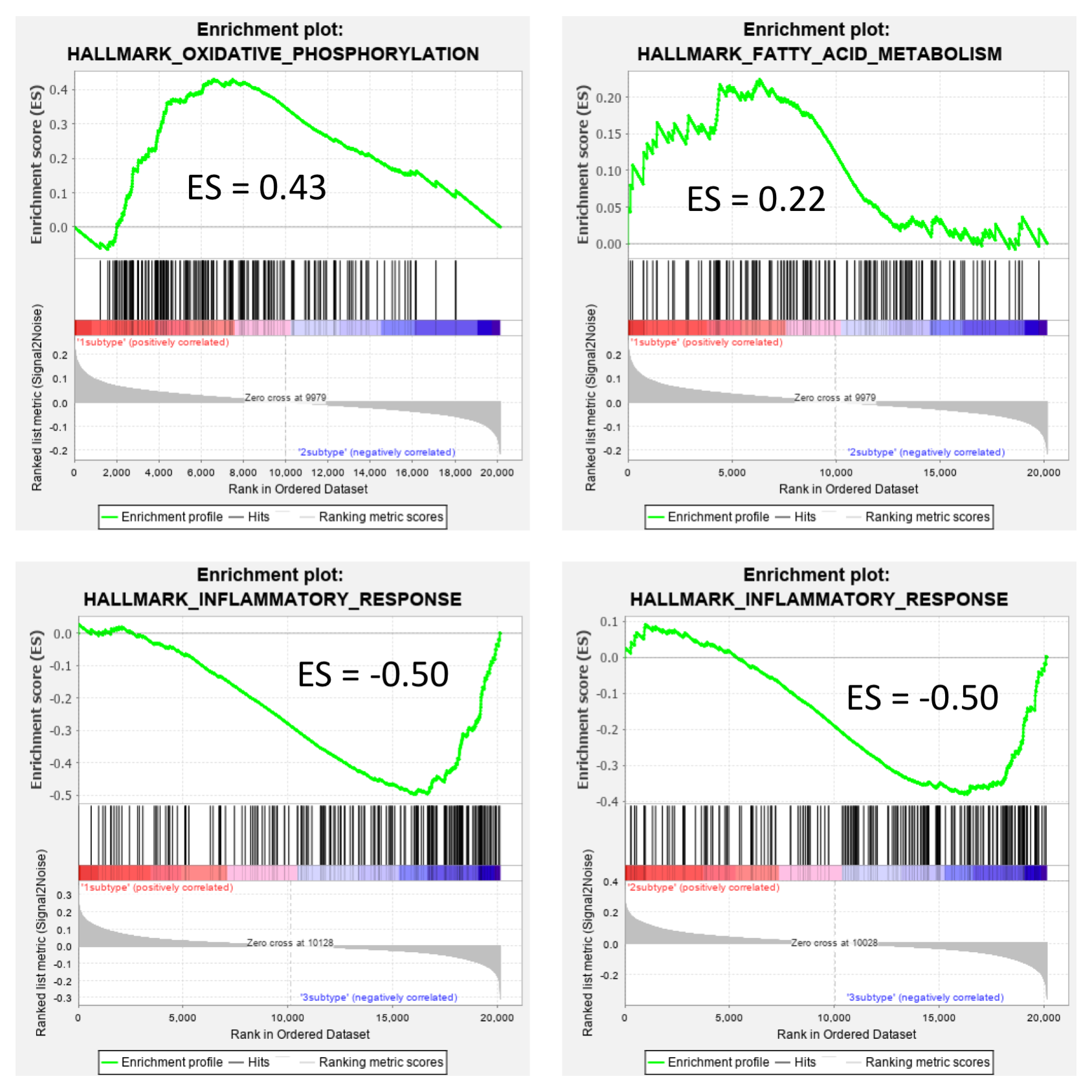

Supplement: Supplementary file 1 [file cancers-14-04035-s001.zip › Supplementary Figure S4.tiff]

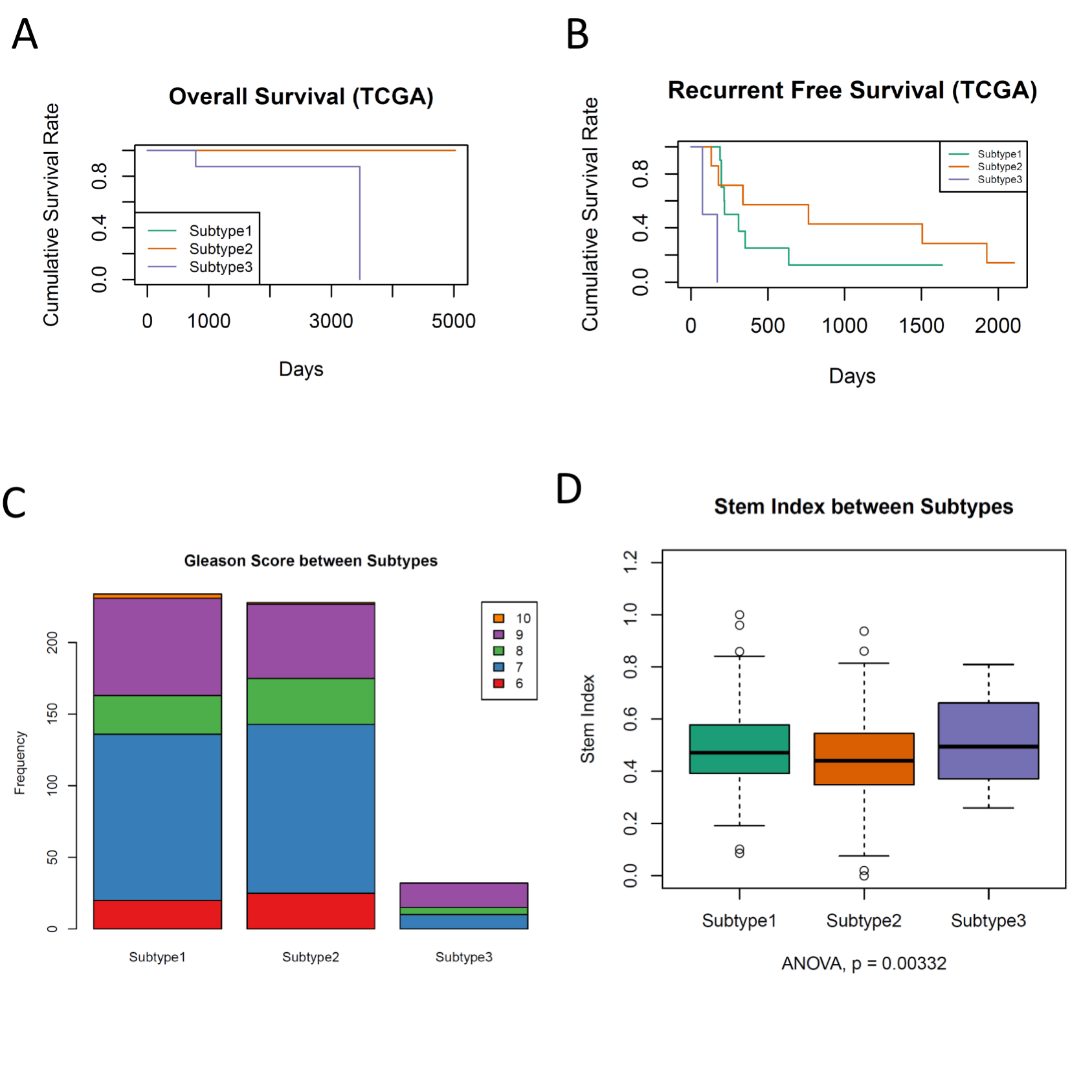

Supplement: Supplementary file 1 [file cancers-14-04035-s001.zip › Supplementary Figure S5.tiff]

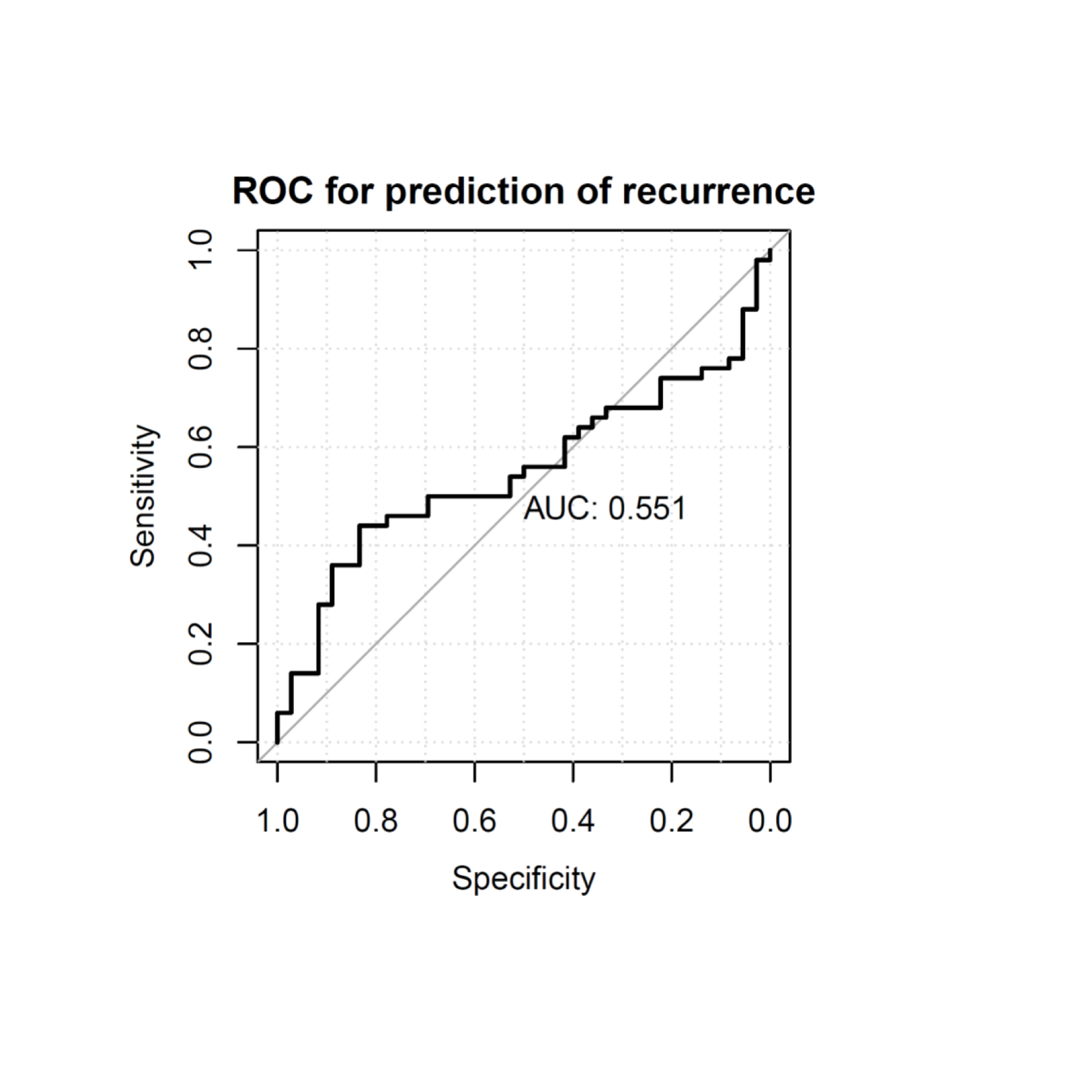

Supplement: Supplementary file 1 [file cancers-14-04035-s001.zip › Supplementary Figure S6.tiff]

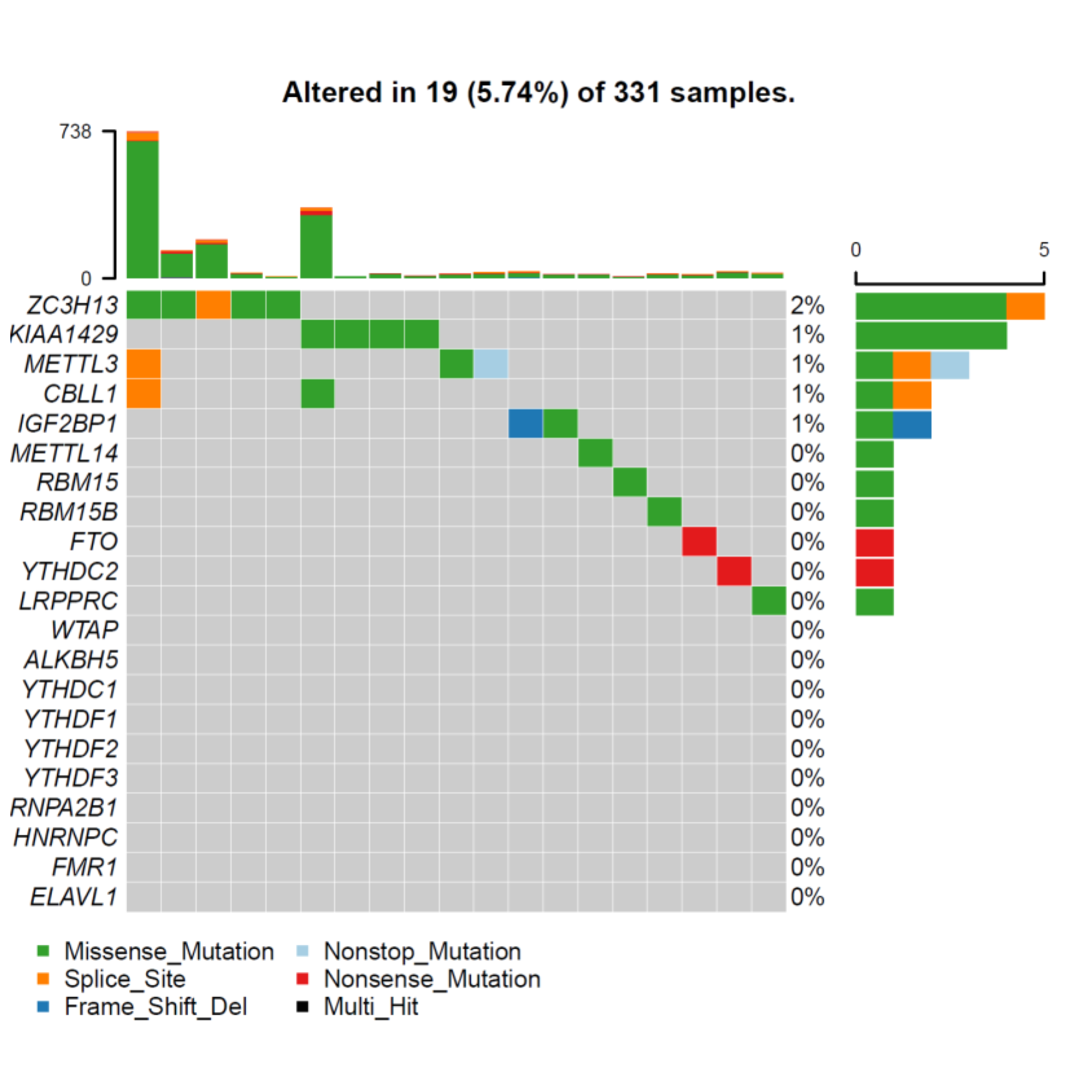

Supplement: Supplementary file 1 [file cancers-14-04035-s001.zip › Supplementary Figure S7.tiff]

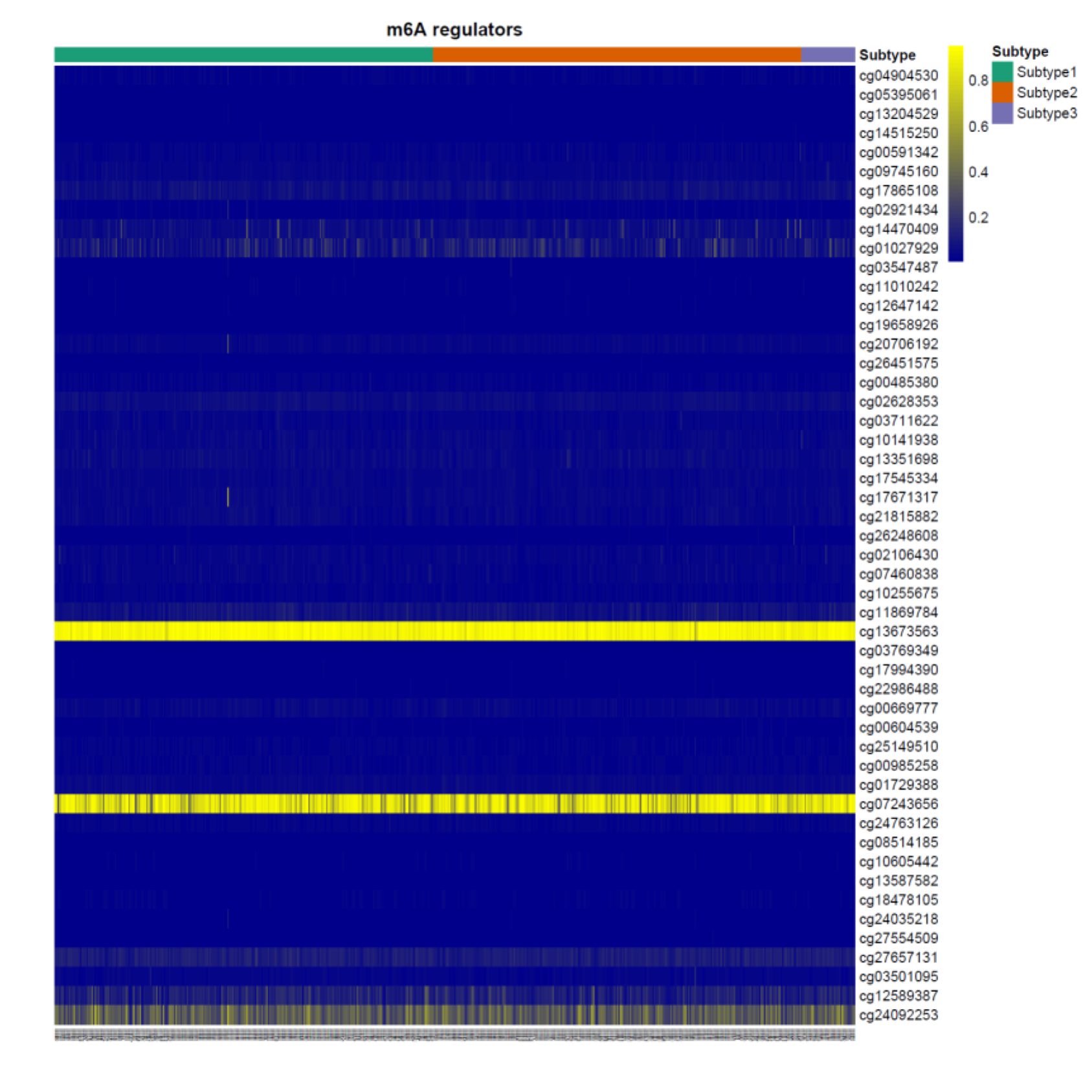

Supplement: Supplementary file 1 [file cancers-14-04035-s001.zip › Supplementary Figure S8.tiff]

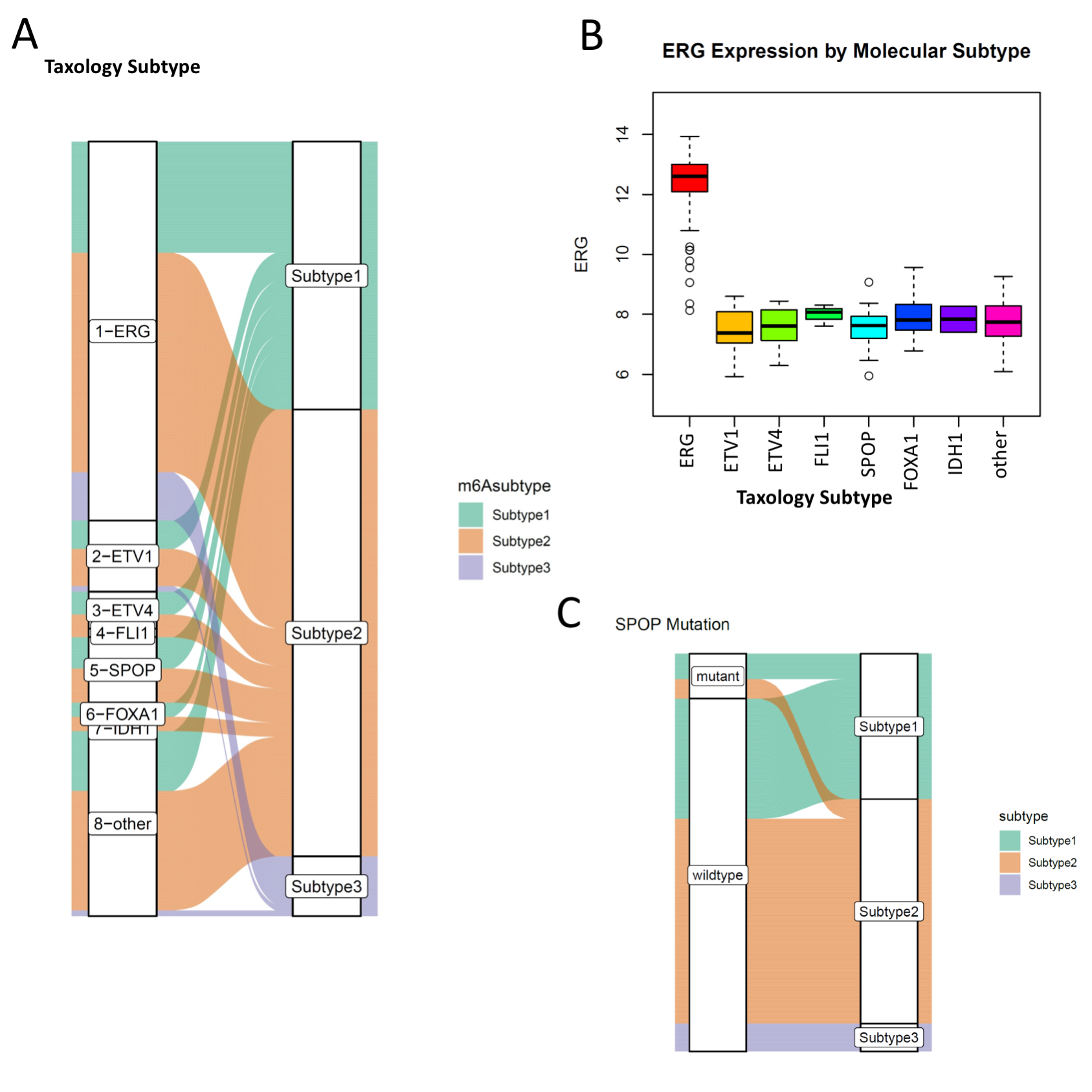

Supplement: Supplementary file 1 [file cancers-14-04035-s001.zip › Supplementary Figure S9.tiff]
